# Push-Pull-Mooring Analysis of Massive Open Online Courses and College Students During the COVID-19 Pandemic

**DOI:** 10.3389/fpsyg.2021.755137

**Published:** 2021-12-09

**Authors:** Kebiao Kang, Ting Wang, Shihao Chen, Yu-Sheng Su

**Affiliations:** ^1^College of Science and Technology, Ningbo University, Ningbo, China; ^2^Department of Computer Science and Engineering, National Taiwan Ocean University, Keelung, Taiwan

**Keywords:** MOOCs, COVID-19, push pull mooring, PLS-SEM, task-technology fit

## Abstract

The partial least squares structural equation modeling (PLS-SEM) provides researchers with an analysis tool for prediction theory. As the coronavirus disease 2019 (COVID-19) brings risks to teaching and learning, students have been forced to switch from classroom learning to online learning and most subjects have chosen massive open online courses (MOOCs) for online learning in China. This study examines whether MOOCs can replace traditional classroom education and explores the factors that influence the intentions of switching of the students from offline to online. We sequenced the PLS-SEM analysis of data with 397 students from a university in Zhejiang province of China, testing the model parameters, and discussing the push-pull-mooring (PPM) theory. Our data demonstrate that security risk is a push factor, switching costs are a mooring factor, and perceived usefulness and task–technology fit are pull factors that pull students from traditional, offline learning to MOOCs. In addition, the PPM model of the analysis results provides a more specific understanding of the importance–performance analysis of each factor. Our findings suggest that to constantly improve the switching intention to address unexpected challenges in the future, teachers should establish an effective emergency management measures, including curriculum design, to be consistent with their needs.

## Introduction

Massive open online courses (MOOCs) represent essential technological advances that have taken place in higher education over the past 10 years. Smart devices and the Internet can be used to participate anytime and anywhere in open and massive online courses designed and provided by accredited higher education institutions and organizations, resolving the educational inequality in traditional teaching. Moreover, the massive online learning resources of such courses help to diverse groups, including elementary and middle school students, college graduates, and professionals, to discover and develop their technological potential, thereby enabling them to respond more favorably to market needs (De Notaris et al., 2021). Educators can upload prerecorded courses on MOOC platforms (and materials, text, audio, and video) and the participatory and interactive experience can be enhanced by combining other social media applications for experience sharing (Alhazzani, 2020; Lee et al., 2021; Su and Lai, 2021). E-learning and digital cultures have increased following the rapid expansion and scale of global campus education. Such interactivity allows learners to undergo interactive learning in a digital environment, even without a specific school or educational institution. Godwin-Jones (2014) proposed that MOOCs enable individuals to learn any course from the best universities through the Internet. In addition, the individuals can learn on MOOC platforms without spatial and temporal limitations (Waldrop, 2013; Shah, 2015).

Moreover, the adoption of the online teaching model has increased due to the coronavirus disease 2019 (COVID-19) pandemic, which has led to the shutdown of educational institutions worldwide. In response to rapid spread of the COVID-19, the Ministry of Education (MOE) of China announced the launch of online teaching in colleges and universities as a measure to reduce infections by reducing contact. The ministry also implemented the policy of “stopping class without stopping learning” to shift the teaching mode from traditional classroom education to online teaching (Zhou, 2021). With the increased popularity of online teaching modes, MOOCs have emerged as one of the online teaching models in China with a high usage rate.

However, the implementation of MOOCs in online teaching in China is not optimal. Castillo et al. (2015) indicated that technology access is the central barrier to MOOC use in developing countries. Insufficient technology access to information and communication technology (ICT) equipment and the limited Internet penetration rate and connection speed have led to challenges in the sustainability of MOOC learning in the long term. MOOC learning in China, as a developing country, is affected by these factors. In addition, considering the factors of knowledge access and education and technological systems, resource distributions in different regions are unequal, which inevitably affect the usage rate of MOOCs to a certain extent. Deng et al. (2019) focused on the design of MOOCs to determine how to optimize the MOOC teaching environment to reduce the pedagogic imbalance caused by social inequality resulting from differences in age, gender, education level, and cultural background. Moreover, Cheng (2019) pointed out that MOOCs can provide a better learning effect than other online learning modes. However, whether it is better than the traditional teaching mode has not yet been discussed.

Massive open online courses can replace traditional teaching styles that have become a matter of controversy, particularly during the pandemic when individuals have had to adjust previous teaching forms owing to the COVID-19. Specifically, many studies conducted during this period examined whether a positive attitude toward continuous learning (and considering it conducive to user development in the face of the pandemic) also plays an important role in the continuous promotion of the online learning mode (Daumiller et al., 2021). The rapid shift from offline teaching to MOOCs as an emergency response to the COVID-19 prompted teachers to focus on solutions for the lack of information technology (IT) ability when responding to the teaching emergency. Finally, they found an appropriate online mode in MOOCs. As a result of the COVID-19, the MOE of China. (2020) stated that China ranks first globally in terms of the number of MOOCs and viewers. Furthermore, MOOCs have become a key factor in promoting higher education reform in China. They not only help Chinese universities to successfully cope with the teaching difficulties under the COVID-19, but also provide valuable experiences in building a high-quality national lifelong learning model. In the future, the MOE of the People’s Republic of China (PRC) will continue to increase investment in the resource construction of MOOCs. As MOOCs have been frequently used during the COVID-19 pandemic, it appears to have replaced the traditional learning mode of college students. During the initial spread of the COVID-19, MOOCs were used very often and appear to have been used instead of the traditional learning mode principally in college students. Giving up an original behavioral habit for a particular reason, referred to as the push-pull-mooring (PPM) theory, was mainly adopted in previous studies to explore changes in behavior modes (Moon, 1995). The essence of the theory is to probe into the modes of user migration behavior. Before the outbreak, students who used MOOCs did not have perfect conditions in terms of technology requirements; however, the reverse applies during the epidemic period. The push of negative influences such as environmental factors, the pull force of the benefits to the positive behaviors of the individuals, and the mooring force of individuals themselves could be seen (Hou et al., 2011; Lai and Chou, 2017). In this process, the environmental safety of MOOCs by students was the push and Chen and Keng (2019) exemplified this by the intentions of the students to transition from face-to-face English classes to online learning platforms. Furthermore, they proposed that to improve the learning efficiency of the students, greater emphasis should be placed on the demands of learners in online learning. Therefore, in this study, we examined motivation of the college students, perceived usefulness, and increased MOOC utilization. Due to the impact of the COVID-19 pandemic, intentions of the students to migrate from offline to online learning have become a crucial challenge in explaining migration behavior under emergency management. According to the debate, this study provided a mode to explain the transition to MOOCs based on the PPM theory, bringing fresh insight for future MOOC advocacy.

Based on the above background, the following speculation can be obtained: Environment, technology, and learning intentions of the students might be the motivating factors for people to suddenly use MOOCs on a large scale. The effects of these factors were explored by PPM to obtain the conclusion that, if there is no pandemic in the future, information technology is not improved as in the past, and offline learning motivation of the students is greater than the online learning motivation, the users of MOOCs may be greatly reduced. Subsequently, the previous large-scale promotion and investment of our country may be wasted.

## Theoretical Framework

The partial least squares structural equation modeling (PLS-SEM) helps researchers to understand the factors that predict the intention of the students to use MOOCs. Moon (1995) discussed the push-pull model, added the mooring factor, and integrated them into the PPM theory. As Moon explained, the push, pull, and mooring factors directly affect the migration factors of the individuals. Moreover, among the factors affecting migration, the concept of mooring probably served as a factor in promoting or hindering migration. Subsequently, the theory was adopted by scholars in different fields from various perspectives and the concept of PPM was explained as consumption cost of the users to explain the impact of their behavior migration (Chen and Keng, 2019). However, PPM differs from the established research constructs of the past in that the theory can efficiently explain various characteristics under various environmental influences according to disparate research situations (Chen and Keng, 2019). Previous studies employed the Technology Acceptance Model (TAM) (Ashrafi et al., 2020; Eksail and Afari, 2020) and the Unified Theory of Acceptance and Use of Technology (UTAUT) (Tseng et al., 2019) to explain the online learning of the students. However, these studies tend to explain the reasons of use of the users at the technical level. Through PPM, the influencing factors for use of the users under different topics can be determined only by considering the uniqueness of backgrounds of the users at that time (Lin et al., 2021).

**FIGURE 1:**
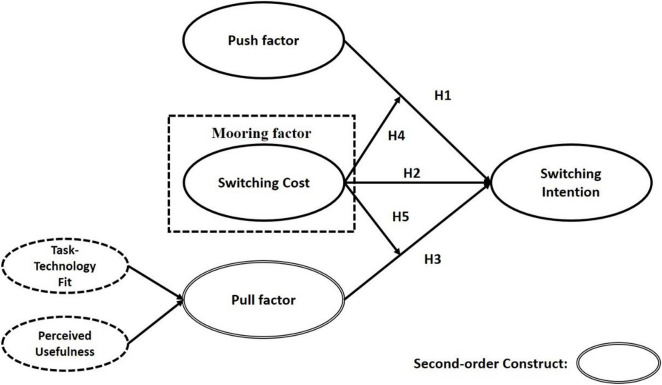
Research model.

This study extends the concept of population migration and investigates the factors affecting switching intention of the students (i.e., feelings of the students as they shift from the traditional, offline mode of classroom learning to MOOCs platforms). By switching learning modes, variables such as perceived usefulness and switching cost proposed by Chen and Keng (2019), and the security risk put forward by Cheng et al. (2019), are incorporated into the model. In addition, task–technology fit (TTF) was extended as an independent variable in this study. Accordingly, a research model was built that can influence the switching behavior of the students as the learning modes change under the impact of the COVID-19 in Chinese colleges and universities. The three factors of “push, pull, and mooring” were defined in accordance with an online learning environment mode to better analyze the reasons for the change of learning modes of the students. Figure 1 illustrates the relationship between the three variables according to the concept of PPM and individual willingness.

### Push Factors

Push factors are factors that push individuals away from their original positions. Security risk was characterized by Cheng et al. (2019) as consumers migrating to alternatives with lower safety risks because of their impression of high security risk with the services they previously used.

Bozkurt and Sharma (2020) proposed that other unpredictable changes such as wars, regional conflicts, natural disasters, and other catastrophes (including the COVID-19 pandemic) affect the learning of the students; thus, remote learning systems are necessary to implement distance education. The benefits of MOOCs, which enable distance education, have been demonstrated for coping with natural disasters. For example, in the COVID-19 pandemic, learning environments of the students have shifted from outdoors to their homes on their personal laptops. Song and Song (2021) found that in South Korean universities with distance education implementation, a comfortable educational environment is one of the reasons for intention of the students to adopt the online learning model; to a certain extent, learning in the home environment can provide personalized space. In a previous study, Earthman (2002) investigated traditional face-to-face classrooms in schools or training institutions of the past; overly complex learning environments or crowded schools or classrooms produced various problems and, thus, affected academic performance of the students. It is evident that students would choose to stay away from traditional, offline courses to avoid security risks during the COVID-19 pandemic when they realize that such risks stem from the external environment. Thus, a push for MOOCs can be created. Therefore, this study proposed the following hypothesis:

H1: The higher the perception of the impact of the university students on safety risks, the lower their acceptance of learning in physical courses and the higher their intention to switch to MOOCs.

### Pull Factors

Pull factors attract individuals to a new role. For example, factors promoting students to switch from the traditional learning model to the model based on MOOC learning platforms are considered as pull factors. Zhenghao et al. (2015) indicated that MOOCs enable users to gain professional benefits in specific forms such as practical skills for earning a promotion, seeking a job, or starting a business. MOOCs can also help to realize self-development-oriented education or educational benefits. Kasch et al. (2021) stated that reasons for the increasing adoption of MOOCs included the immediate availability of the materials, free selection of the students, and continuous learning enabled by the course arrangement. These factors were related to the perceptions of the students of MOOC usability, which also indirectly affected the feedback between students and their peers (Julia and Marco, 2021). This demonstrates that MOOC usability could be a reason for increased user adoption.

This study focused on the TTF model proposed by Goodhue and Thompson (1995). This model explained the capabilities of IT in supporting job tasks and determined the effects of IT on individual task performance by describing cognitive psychology and cognition behavior, thereby reflecting the logical relation between IT and task requirements (Goodhue and Thompson, 1995). Relevant research has focused on how attributes of IT, such as quality, ease of use, and usefulness, affect the attitude of the users, emotions, beliefs, motivation, and self-efficacy regarding the information system (Tao and Xu, 2012). In previous studies, TTF was not mentioned in the push-pull theory (Khan et al., 2018). Wu and Chen (2017) indicated that TTF was rarely applied in online learning; in particular, no specific investigation has been conducted regarding whether MOOC usage of the students has positive effects on their learning outcomes. However, Khan et al. (2018) used TTF as a theoretical framework to predict the acceptance of MOOCs by students in developing countries. They concluded that TTF had positive effects on behavioral intention. Goodhue and Thompson (1995) showed that users could perceive their daily tasks such as learning as simple and effective through TTF. Therefore, if MOOC technology fits the tasks of the individuals, the acceptance and adoption rates of MOOCs would inevitably increase. This study suggests that during the COVID-19 pandemic, learners identified the usefulness and TTF of online MOOC platforms. Therefore, students are more willing to accept the change from traditional offline mode to MOOC platforms. Overall, the main factors that influence the pull force proposed in this study include usefulness and TTF, which serve as positive factors that drive the change. Based on the discussion above, this study proposes a second hypothesis:

H2. The higher the perceived switching costs of university students, the lower their intention to switch from physical courses to MOOC learning.

### Mooring Factors

The mooring factors in this study referred to the negative factors that hindered individuals from switching from the traditional environment to the online environment. Switching costs are regarded as a crucial mooring factor; they are important explanatory variables for switching behavior regarding platforms (Xu et al., 2014; Chang et al., 2017; Jung et al., 2017; Cheng et al., 2019) and a decisive factor that determines and regulates customer satisfaction (Burnham et al., 2003). Switching costs are the costs incurred by a user as a direct result of switching from the *status quo* to a new situation (Cronin et al., 2000). Moreover, the concept of switching costs varies among different situations. Klemperer (1995) indicated that such costs not only affect switching behavior of the customers, but also provide a competitive advantage. Nilssen (1992) divided switching costs of the users into transaction costs and learning costs. Fornell (1992) indicated that such costs implied costs for searching materials, discounts of the customers, financial conditions, habits of the customers, emotional costs, cognitive effort, and psychosocial risks. Therefore, in the service industry, perceptions of the customers of switching costs should be considered from the perspective of highly customized and personalized services and geographical distribution (Jones et al., 2007).

Accordingly, given the current popular trend of online learning in China, relevant switching costs for MOOCs should be considered to maintain long-term satisfaction of the students and to prevent their switching to other learning modes because of past habits and emotional costs. Given personalized services and associated discounts of MOOCs for learners, MOOCs provide, in terms of cost, a practical alternative to expensive face-to-face training courses (Young et al., 2017). However, according to Porter (2015), the investment cost for MOOCs differs among universities and depends on a series of factors including the subject area, mode of learning, type of materials used, and level of experience of the development team. Moreover, additional fees are required in courses involving specific learning designs; therefore, the budget should be increased if open educational resources (OERs) do not apply. In other words, students did not need to pay additional costly fees if the instruction material in MOOCs was paid by the school or if OERs were sufficient for MOOCs to meet the needs of the students. Thus, in this study, the third hypothesis is proposed:

H3. The greater the impact of perceived usefulness and TTF perceived by university students, the higher the intention to switch to MOOC learning.

With respect to switching costs, such learning costs also involve the pattern of habitual behavior and emotional cost of an individual. Ma and Lee (2020) examined customers who used MOOCs in the least developed countries in Asia and found that traditional teaching and peer influence were the main obstacles to MOOC usage. Thus, the drive for customers, or “residents”, to stay in, or leave, their original place of residence can be summarized as mooring, an influential concept on migration behavior (Moon, 1995). Thus, among factors that hinder the adoption of MOOCs, the traditional teaching mode is a reason affecting MOOC usage of the students. Krauth (2015) indicated that, ultimately, MOOCs do not facilitate one-on-one and face-to-face relational services between teachers and students. In addition, the cost of learning during the transition from traditional, offline learning to online learning environments is considered as a key factor affecting learning efficiency of the students (Chen and Keng, 2019).

Given their own habits and preferences, individuals may be unwilling to switch to new and more favorable services (Gefen et al., 2003; Grewal et al., 2003). In the past, students in China used to learn exclusively in offline classrooms. However, during the COVID-19 pandemic, learning through MOOCs at home prevents risk to the COVID-19 exposure outdoors. Thus, during the COVID-19 pandemic, the most important consideration in MOOCs is safety. Polites and Karahanna (2012) showed that switching behavior occurs when alternative services provide greater benefits than those of the original services. Therefore, during the COVID-19 pandemic, students switched to MOOCs if these provided greater benefits than traditional teaching. Lin et al. (2021) regarded this learning efficiency produced from switching learning platforms of the students during the COVID-19 pandemic as a mooring factor for their continual use of online teaching and relevant personal habituation was also considered. Thus, considering the learning cost involved in switching from traditional learning to MOOCs as the impact of mooring on driving factors, the fourth hypothesis is proposed as:

H4. The poorer the relationship between the push factors and the switching intentions of the students, the higher the switching cost.

In addition to the factors involved in learning costs, the cost of searching for materials plays a crucial role. Covach (2013) indicated that although MOOCs may not replace traditional university education, much effort has been made to provide MOOCs as free courses for knowledge dissemination to a wide range of individuals. This implies that MOOCs with free, rich, and diverse materials would ensure continuous usage of the students of MOOCs. Thus, the fifth hypothesis is proposed:

H5. The weaker the relationship between the pull factors and the switching intentions of the students, the higher the switching cost.

## Methodology

### Construct Operationalization

The perceived security risk arose from the three questions raised (Grewal et al., 2003). In terms of operational definition, the perceived security risk is defined as a safety problem that affects the offline courses during the COVID-19 pandemic, which prompts college students to turn to the online learning platform with a lower security risk (Lin et al., 2021). Second, the pull factor consists of two subconstructs of usefulness and TTF. Gefen et al. (2003) suggested that the variables of perceived usefulness were evaluated by using two scales. In terms of operational definition, perceived usefulness is defined as the feeling that the online platform helps them to learn and use. The four tasks technology adaptation was taken as the research object (Isaac et al., 2019). In terms of the operability of questionnaire design, whether the learning objectives and online platform meet the needs of learning of the students were mainly explored. To ensure the accuracy of the analysis of measurement items, in this study, based on the operational definition of the pull factor by existing studies, the construct is defined as the formative indicator of a two-stage model consisting of two subconstructs including TTF and usefulness (Chen and Keng, 2019; Lin et al., 2021). The switching cost and switching intention were designed with the scale introduced by Chen and Keng (2019) and Jin et al. (2021). In terms of operational definition, the switching cost refers to the loss of learning efficiency of the students when they switch from the offline to online learning platform during the COVID-19 pandemic. Meanwhile, the switching intention refers to the willingness to switch from the offline to the online learning. All the items were measured by the Likert 7 subscale. The questionnaire was designed mainly based on the original English scale. Since the main subjects of this study were Chinese university students, the questions of the questionnaire were translated into Chinese by the three university professors and then through reverse translation, the concepts of the translated questions were confirmed to be no different from those of the previous ones.

### Data Collection

In this study, before the formal questionnaire survey was conducted, preliminary pretesting of the questionnaire was performed and relevant scales in the literature were compiled. This was followed by a simple trial test with participants from the university where the researcher worked with the total sample comprising 32 students from a class. Through a trial test, the design of the research questions was verified. With respect to the distribution of the questionnaires, the survey was conducted exclusively online, as the COVID-19 pandemic was still at its peak. The main reasons for selecting online questionnaires include the following: First, numerous universities have adopted online courses due to the COVID-19 pandemic; thus, a large number of university students are studying at home. Therefore, survey samples can be collected effectively by adopting online questionnaires. Second, online questionnaire collection could reduce the cost of questionnaire recovery, shorten the time for questionnaire responses, and questionnaires could be provided to appropriate users (Bhattacherjee, 2002; Su et al., 2021).

Therefore, data from the formal questionnaire were gathered through Wenjuanxing.^1^ The survey period was from late May to mid-June 2020 (when courses of the students were about to end) following a semester-long experience of using MOOCs. As the researcher adopted convenience sampling to ensure the effective distribution of the questionnaire, respondents were mainly students from a university in Zhejiang province. The questionnaire was distributed to a teacher group on WeChat and group members were asked to help forward the questionnaire to students participating in MOOCs and to encourage the students to answer voluntarily. The main objective of the research questions was to examine the switching behavior of university students from courses in traditional physical classrooms to MOOCs due to the COVID-19 pandemic. A total of 403 valid questionnaires were returned and 6 invalid questionnaires were excluded by removing invalid samples (the criteria for judging invalid questionnaires included excessively extreme responses [(such as 1 and 7) or uniform responses for all the items]; the number of valid questionnaires was 397.

The study population comprised 111 male (27.96%) university students and 286 female (72.04%) university students. Students who had never used MOOCs before the COVID-19 pandemic accounted for 57.49%, whereas those who had used MOOCs to study before the COVID-19 pandemic accounted for 42.51%. Finally, in terms of the distribution of respondents by colleges, 173 respondents were from the school of business/school of management, accounting for 43.56% of all the respondents, followed by 91 (22.37%) respondents from the school of humanities and the school of law, 81 (20.84%) respondents from the school of science and engineering, and 53 (13.23%) respondents from the school of education.

## Results

First, the measurement model analysis included the Cronbach’s alpha validity, convergence validity, and discriminant validity, which verified the reliability and validity of the scale measurement. Second, the PLS was used to evaluate the model. The PLS-SEM was chosen for analysis, as it was more suitable than SEM based on covariance and was mainly adopted to conduct an exploratory study of theoretical development and the potential variable scores were required for subsequent analysis in this study (Balakrishnan et al., 2017). In addition, a second-order model with a reflective form was adopted. The pull factor is a second-order forming entity in this study with pull factors including a reflective construct dimension-perceived usefulness and TTF. However, because SEM based on covariance cannot test the second-order formative construct, the PLS-SEM is the best option for testing the second-order formative construct (Balakrishnan et al., 2017). As a result, the PLS approach was appropriate for model analysis in this study.

The common method variance (CMV) has two solutions. First, in the questionnaire design, the questionnaire items were paged to reduce the weariness of the respondents caused by long content when answering the questionnaire and to reduce the influence of CMV by continuous answering. Second, the Harman single factor test was used to check whether CMV was available. Principal component analysis (PCA) was adopted in the experiment and through the test results. It could be determined that there was no single factor accounting for more than 50% of the impact and the factor explained shows that the value is 0.385. Based on the test results, no CMV occurred in this study and all the results were in line with the values recommended in a previous study (Balakrishnan et al., 2017). Finally, Hair et al. (2017) suggested that the variance inflation factor (VIF) should be below 5 for this study. The research results showed that all the index values ranged from 1.573 to 3.685; therefore, it is expected to be in line with good results as shown in Table 1.

**TABLE 1:** Research topics on the push-pull-mooring (PPM) theory.

Construct	Items	Factor loading	α	CR	AVE	VIF
Perceived security risk (SER)	SER1 SER2 SER3	0.966*** 0.960*** 0.943***	0.954	0.970	0.915	1.573
Perceived usefulness (PU)	PU1 PU2	0.964*** 0.965***	0.925	0.964	0.931	3.685
Task-technology Fit (TTF)	TTF1 TTF2 TTF3 TTF4	0.956*** 0.959*** 0.946*** 0.901***	0.957	0.969	0.885	3.685
Switching Cost (SwiCo)	SwiCo1 SwiCo2 SwiCo3	0.904*** 0.880*** 0.873***	0.864	0.916	0.785	1.951
Switching intention (SW)	SW1 SW2 SW3 SW4	0.917*** 0.946*** 0.898*** 0.932***	0.942	0.959	0.853	DV

***p < 0.05.

### Measurement Model

In terms of the results from measuring convergence value, Hair et al. (1998) proposed that convergence value was mainly used to measure the composite reliability (CR) and average variance extraction (AVE) among various constructs. According to the sample results recovered by respondents, component reliability, the Cronbach’s alpha, and AVE results listed in Table 1, the CR values between the measurement constructs in this study ranged from 0.864 to 0.957, all higher than the recommended values (CR value was higher than 0.7) proposed by Hair et al. (2017). As such, these constructs are internally consistent. Moreover, the AVE of the construct itself is also higher than previously suggested values by more than 0.5 (Yuan and Powell, 2013), indicating that this result has good convergence value. The AVE of the construct ranged from 0.785 to 0.931, which is consistent with the recommendations in previous studies. Finally, the second-order formative indicator is assessed according to whether its weight is significant (*p* < 0.05) (Wang and Haggerty, 2011; Lin et al., 2021). Based on the results in Table 1 below, the weight values of the two dimensions, perceived usefulness and TTF, of the pull factor show a significant influence, so that these two indicators can form the concept of the pull factor.

Discriminant validity refers to the degree to which an indicator of a potential variable differs from other indicators of potential variables that form part of the model (Balakrishnan et al., 2017). Two criteria were used to evaluate the discriminant validity. Traditional, discriminative validity measures include the observation of cross-loading, as it requires that the variance of an underlying variable with its related indicators be greater than that of other potential variables in the model (Yuan and Powell, 2013). In addition, traditionally, the existence of discriminant validity is confirmed when the square root of the AVE of each construct is greater than the correlation between the related construct and all other constructs (Yuan and Powell, 2013), as shown in Table 2. However, recent studies have proposed replacing traditional measures with the heterotrait-monotrait correlation ratio (HTMT) as an alternative method for assessing discriminant validity (Henseler et al., 2014). Henseler et al. (2014) suggested 0.90 as a threshold value for structural models with constructs. In this study, the values ranged from 0.492 to 0.856, indicating that discriminant validity was established for all the constructs of the model, as shown in Table 3.

**TABLE 2:** Analysis of discriminant validity (Fornell–Larcker Criterion).

	TTF	SER	PU	SW	SwiCo
TTF	**0.941**				
SER	–0.470	**0.957**			
PU	0.804	–0.492	**0.965**		
SW	0.776	–0.558	0.766	**0.924**	
SwiCo	–0.559	0.625	–0.562	–0.676	**0.886**

*SER, perceived security risk; SwiCo, switching cost; TTF, task–technology Fit; SW, switching intention; PU, perceived usefulness. The bold values indicate average variance extraction (AVE) square root.*

**TABLE 3:** Analysis of heterotrait-monotrait.

	TTF	SER	PU	SW	SwiCo
TTF					
SER	0.492				
PU	0.856	0.524			
SW	0.817	0.589	0.820		
SwiCo	0.614	0.684	0.626	0.745	

*SER, perceived security risk; SwiCo, switching cost; TTF, task–technology Fit; SW, switching intention; PU, perceived usefulness.*

### Structural Model

**FIGURE 2:**
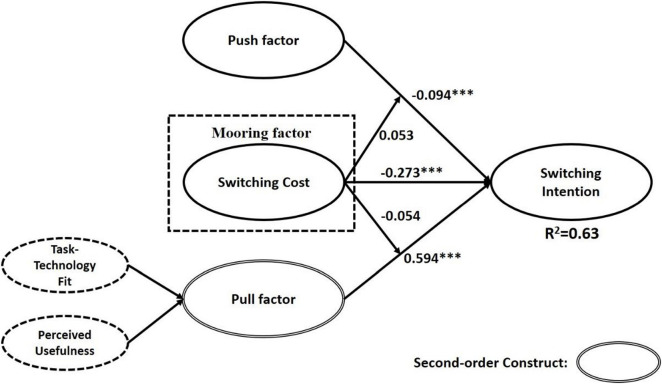
Research analysis result. ***p < 0.05.

The H1–H5 parts were mainly analyzed in this study based on the results. According to the statistical analysis results, the overall explanatory value of this study was as high as 0.63, which implies a high overall explanatory value in this study. Based on H1 results, perceived security risk (β = –0.094, *p* < 0.05, *t* = 2.040) had a negative influence on switching intention. Second, based on the H3 result, the pull factor (β = 0.594, *p* < 0.05, *t* = 10.047) had a significant positive impact on switching intention. Third, the switching cost of H2 (β = –0.273, *p* < 0.05, *t* = 4.520) also had a significantly negative relationship with the switching intention. Finally, in terms of the influence of the adjustment effect, the results showed that neither H4 (β = 0.053, *p* > 0.05, *t* = 1.334) nor H5 (β = –0.054, *p* > 0.05, *t* = 1.812) habits have an adjustment effect on the push/pull factors. The results of this research model are summarized in Figure 2.

## Conclusion and Discussion

### Conclusion

The empirical results revealed that security risk was the push factor that transferred students from traditional, physical classrooms to MOOC platforms. During the COVID-19 pandemic, students felt insecure and preferred not to attend classes in physical classrooms, which corresponded with the switching intention demonstrated by previous PPM research (Cheng et al., 2019; Chen and Keng, 2019). In terms of the push factor, security was a key indicator that drove students out of physical classrooms (Cheng et al., 2019). Because of the COVID-19 pandemic, students were concerned about the lack of security measures in traditional, physical classrooms and preferred to take courses on MOOC platforms. Considering the difficulties of conducting traditional, in-person courses during the COVID-19 pandemic, teachers should establish effective emergency management measures (e.g., an emergency security mechanism and a valid approach to efficiently implement online courses and establish new ones) to address unexpected challenges in the future. However, when the threat of COVID-19 is over and students feel safer outside, their learning habits may switch from online to offline because of reduced security risks.

In terms of pull factors, the use of MOOC platforms during the pandemic was affected by two essential subdimensions, namely perceived usefulness and TTF. Studies have indicated that TTF is the key dimension influencing intention of the students to engage in online learning (Khan et al., 2018; Isaac et al., 2019). Students might complete their education and learning tasks through MOOCs during the COVID-19 pandemic. Thus, the key to increasing goodness of fit of the students is the selection of teachers and schools on MOOC platforms and compatible teaching materials. Students are more likely to accomplish their learning tasks and engage in MOOC learning when the design of an online course is consistent with their needs. In addition, perceived usefulness affects the intention of the students to transfer to MOOC learning. This factor is consistent with the findings on online learning switching intentions in the literature (Balakrishnan et al., 2017; Cheng et al., 2019). After the COVID-19 pandemic, universities are advised to provide more diverse MOOC content and develop personalized materials to attract students to MOOC platforms such as enabling students to make their selections on teachers and instructional resources for MOOC platforms. For instance, when establishing a new course, a teacher can record relevant MOOCs and incorporate regional instances into lessons. The course can thus become more aligned with local needs and enable students to provide feedback on the course content during online face-to-face interactions. Furthermore, MOOCs offer many out-of-school curriculum resources, and to a certain degree, supplement the study of resource distributions across the different regions that may be unequal.

According to the research results, the cost of switching traditional, physical courses into MOOCs is low, which is consistent with the conclusions of previous studies (Cheng et al., 2019). During the COVID-19 pandemic, students in Chinese universities showed an increased willingness to switch to MOOCs due to their lower cost. Therefore, this study suggests that to constantly improve switching intention, the focus should be on curriculum design, especially the establishment of specific courses in different regions. Local higher schools need to design courses in combination with local industries, where the course content may be added through the integration of industry and learning. The cost and time of MOOC course construction would be effectively reduced as well.

### Implications for Research

This study makes several significant contributions. First, efforts have been made to build barrier-free and high-quality education for MOOCs, which differs from other e-learning courses in universities (Yuan and Powell, 2013). When universities in China adopted emergency response strategies under the COVID-19 pandemic, students switched from the traditional learning mode to online MOOCs. This study determined the factors influencing migration behavior, which differed from the factors influencing daily use of the students of MOOCs in the past. In particular, this study examined the effects of security risks, TTF, and switching costs on PPM switching behavior. The study results enrich the expectations and experiences of MOOCs across different environmental backgrounds (Bagozzi, 2007). The interaction of these three factors also indirectly influenced motivation of the users to use MOOCs and their preferences. Our findings suggest that to constantly improve the switching intention to address unexpected challenges in the future, teachers should establish effective emergency management measures, including curriculum design, to be consistent with needs of the students to adapt to different environments.

### Limitations and Future Research

This study had some limitations. First, it used a questionnaire survey and the number of recovered samples was limited. In particular, the samples were not obtained from universities in all the provinces in China and a single, specific university was surveyed. Therefore, subsequent studies should examine whether, given various regional characteristics, the COVID-19 pandemic affected the promotion of MOOC-based learning and the switching intentions of the students. Second, this was a cross-sectional study. However, under the continuous evolution of the COVID-19 pandemic, longitudinal studies should be conducted to explore and observe the factors of interest at different time points, which can further clarify whether the learning mode that emerged during the COVID-19 pandemic accelerated the subsequent promotion of MOOC platforms in universities. This study also did not discuss whether the basic information data had an adjustment effect on the structure of this study, which remains for further analysis in the future. Finally, this study is still in its initial stage and is unable to obtain a date for a normal environment. However, when possible, we suggest extracting data in a non-epidemic environment in the future.

## Data Availability Statement

The original contributions presented in the study are included in the article/supplementary material, further inquiries can be directed to the corresponding author/s.

## Ethics Statement

Ethical review and approval was not required for the study on human participants in accordance with the local legislation and institutional requirements. Written informed consent for participation was not required for this study in accordance with the national legislation and the institutional requirements.

## Author Contributions

All authors contributed equally to the conception of the idea, implementing, analyzing the experimental results, writing the manuscript, read, and approved the final manuscript.

## Conflict of Interest

The authors declare that the research was conducted in the absence of any commercial or financial relationships that could be construed as a potential conflict of interest.

## Publisher’s Note

All claims expressed in this article are solely those of the authors and do not necessarily represent those of their affiliated organizations, or those of the publisher, the editors and the reviewers. Any product that may be evaluated in this article, or claim that may be made by its manufacturer, is not guaranteed or endorsed by the publisher.

## Footnotes

1
www.wjx.cn
